# Interaction Mode of the Novel Monobactam AIC499 Targeting Penicillin Binding Protein 3 of Gram-Negative Bacteria

**DOI:** 10.3390/biom11071057

**Published:** 2021-07-19

**Authors:** Stefan Freischem, Immanuel Grimm, Arancha López-Pérez, Dieter Willbold, Burkhard Klenke, Cuong Vuong, Andrew J. Dingley, Oliver H. Weiergräber

**Affiliations:** 1 Institute of Biological Information Processing (IBI-7: Structural Biochemistry) and Jülich Centre for Structural Biology (JuStruct), Forschungszentrum Jülich, 52425 Jülich, Germany; s.freischem@fz-juelich.de (S.F.); d.willbold@fz-juelich.de (D.W.); a.dingley@fz-juelich.de (A.J.D.); 2 Institut für Physikalische Biologie, Heinrich-Heine-Universität Düsseldorf, 40225 Düsseldorf, Germany; 3 AiCuris Anti-Infective Cures AG, 42117 Wuppertal, Germany; immanuel.grimm@Aicuris.com (I.G.); arancha.lopez@aicuris.com (A.L.-P.); burkhard.klenke@gmail.com (B.K.); cuong.vuong@aicuris.com (C.V.); 4 Centre for Bacterial Cell Biology, Biosciences Institute, Newcastle University, Newcastle upon Type NE2 4AX, UK

**Keywords:** AIC499, PBP3, β-lactam, antibiotic, penicillin binding protein, monobactam, structure, transpeptidase domain, Gram-negative bacteria

## Abstract

Novel antimicrobial strategies are urgently required because of the rising threat of multi drug resistant bacterial strains and the infections caused by them. Among the available target structures, the so-called penicillin binding proteins are of particular interest, owing to their good accessibility in the periplasmic space, and the lack of homologous proteins in humans, reducing the risk of side effects of potential drugs. In this report, we focus on the interaction of the innovative β-lactam antibiotic AIC499 with penicillin binding protein 3 (PBP3) from *Escherichia coli* and *Pseudomonas aeruginosa*. This recently developed monobactam displays broad antimicrobial activity, against Gram-negative strains, and improved resistance to most classes of β-lactamases. By analyzing crystal structures of the respective complexes, we were able to explore the binding mode of AIC499 to its target proteins. In addition, the apo structures determined for PBP3, from *P. aeruginosa* and the catalytic transpeptidase domain of the *E. coli* orthologue, provide new insights into the dynamics of these proteins and the impact of drug binding.

## 1. Introduction

In 2014, the WHO published an alarming report about the increasing proportion of multi drug resistant (MDR) bacterial strains in human infections, which is insufficiently accounted for by the development of new antibacterials and was thus declared one of the most important public health threats of the 21st century [1]. Originally named after their ability to interact with penicillin, penicillin binding proteins (PBPs) are well established targets of a larger family of antibiotics containing a β-lactam moiety [2]. Their physiological function is associated with the synthesis, maintenance, and remodeling of peptidoglycan (PG), which is a major component of the bacterial cell wall [3]. PG, also known as murein, consists of linear β(1→4)-linked chains of alternating *N*-acetylglucosamine (GlcNAc) and *N*-acetylmuramic acid (MurNAc) units, which are cross-linked via short peptide chains attached to MurNAc [4]. Consequently, synthesis of PG requires glycosyltransferase (GTase) activity for joining GlcNAc-MurNAc disaccharides, which are available as a membrane-anchored precursor (lipid II), to the growing chain, and transpeptidase (TPase) activity for establishing peptide cross-links.

PBPs have been categorized into high and low molecular mass (HMM, LMM) groups. HMM PBPs collectively confer the abovementioned activities and are thus essential for peptidoglycan synthesis, whereas LMM PBPs are mainly involved in maintaining and recycling the PG, mostly acting as endopeptidases or carboxypeptidases [3]. HMM PBPs can be further subdivided into class A and class B PBPs. While class A PBPs are bifunctional proteins, catalyzing both glycosyl transfer and transpeptidation, class B PBPs are monofunctional TPases, although they do also possess one or more additional domains mediating, for example, protein-protein interactions [3,5]. Subclass B3 PBP (known as PBP3 in *Escherichia coli* and encoded by the *ftsI* gene) is active during cell division and localizes to a multiprotein complex termed the divisome, where it functions in PG synthesis in concert with the division-specific glycosyltransferase FtsW [6,7,8,9]. PBP3 consists of three major domains: the *N*-terminal transmembrane helix anchors the protein in the cell membrane, while the *C*-terminal transpeptidase domain (TPd) is the catalytic module of PBP3 and mediates cross-linking of PG chains by ω-peptide linkages between d-Ala and *meso*-diaminopimelic acid (*m*-DAP) residues on opposing peptide chains [10]. The function of the intervening region (sometimes referred to as the non-penicillin binding domain, n-PBd [11,12]) is not well-defined but may be important for protein-protein interactions [13]. The catalytic serine acting as a nucleophile in the transpeptidation mechanism is also able to react with β-lactam antibiotics because of their structural similarity with the d-Ala-d-Ala motif; the resulting covalent intermediate, however, has a very long half-life (with turn-over constant *k*_3_ ~ 10^−4^ M^−1^s^−1^ or less), effectively leading to irreversible inactivation [2]. Because PBP3, involved in cell division, is essential for the survival of bacteria, β-lactams have proven their value as efficient bactericidal agents for many decades and are still the most widely used antibiotics [14,15]. Unfortunately, bacteria have acquired mechanisms counteracting β-lactams, including β-lactamases, efflux pumps, and modified PBPs displaying lower sensitivity to these drugs. Thus, it is important to develop new compounds with improved antibacterial properties, especially for combatting MDR bacteria.

**Figure 1:**
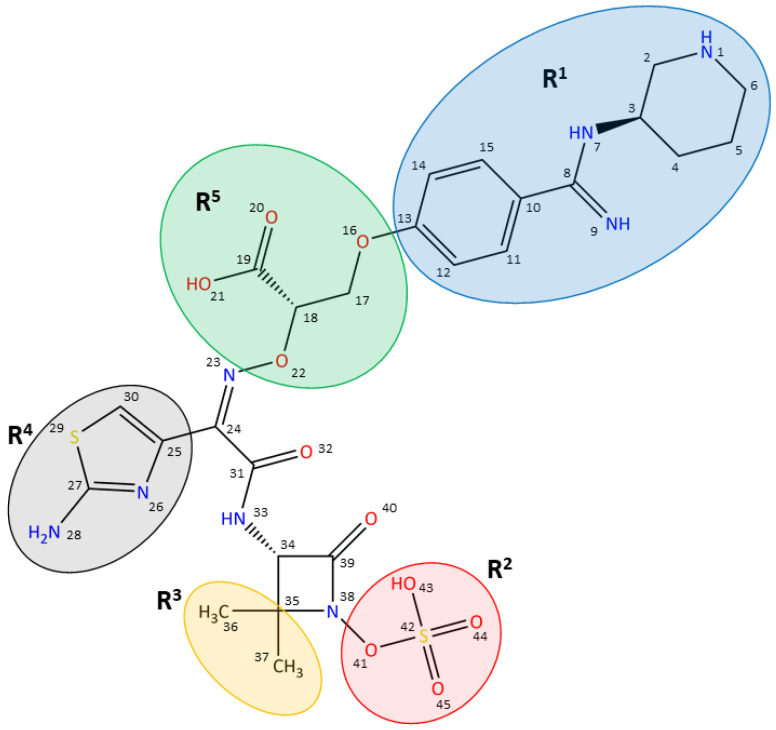
Structure of the monobactam AIC499, obtained by iterative optimization. The relevant functional groups are marked in color: benzamidine-based head group (R^1^), blue; β-lactam N-1 position (R^2^), red; β-lactam C-4 position (R^3^), orange; amino-thiazole (R^4^), gray; linker (R^5^), green.

Basically, β-lactam antibiotics are divided into five classes: penicillins, cephalosporins, monobactams, carbapenems, and penems [16]. Among these, the monobactams represent an underexplored class of marketed antibiotics, with aztreonam being the only representative available worldwide [3]. Aztreonam is based on a natural compound that was chemically modified to increase antimicrobial activity [17,18]. It features high resistance to β-lactamases and strong affinities to PBPs from Gram-negative bacteria, but with its more wide-spread administration, bacterial strains with acquired resistance have been observed [18]. Thus, there is both need and potential for further optimization of this class of β-lactams to target specific bacteria and adapt to varying resistance profiles. LYS228 (originally discovered at Novartis) and MC-1 (a siderophore conjugate developed by Pfizer) are two examples of monobactam drug candidates tackling Gram-negative bacteria such as *Enterobacteriaceae* or *P. aeruginosa,* with LYS228 currently being in clinical phase II [11,19,20,21,22]. AiCuris previously reported on the development of a novel monobactam (AIC499), which shows remarkable inhibitory activity against PBPs of Gram-negative bacteria, and good antibacterial activity, even against clinical isolates harboring several β-lactamase classes [23,24]. During optimization towards AIC499, five important groups were explored to improve the antibacterial potency of the lead structure against *Enterobacteriaceae* and the non-fermenter *P. aeruginosa* (Figure 1). Target optimization was mainly driven by investigating the inhibition of *P. aeruginosa* PBP3 (*Pa*PBP3, unpublished data).

While the amino-thiazole (R^4^) was found to be crucial for potent antimicrobial activity, variation of the R^2^ and R^3^ positions led to an optimum of having two methyl groups on the β-carbon and a sulfate group attached to the nitrogen of the β-lactam ring. The optimal linker (R^5^) length was found to be ethylene, and introducing the carboxylate function modulated the overall antibacterial and ADME profile. Finally, substituting the head group (R^1^) with a piperidine moiety increased antibacterial activity.

In this report, we aim to explore the X-ray structures of covalent intermediates formed with PBP3 from *E. coli* and *P. aeruginosa* to rationalize the binding mechanism of the five functional groups in AIC499. Additionally, we determined the apo structures of *Pa*PBP3 (different space groups) and of a newly designed TPd construct featuring *Ec*PBP3. Besides serving as a reference for investigating the impact of AIC499, these apo structures also provided new insights into various aspects of the structure and dynamics of PBP3 from these two model organisms.

## 2. Materials and Methods

*Cloning, expression and purification.* All PBP3 variants used in this study were expressed as soluble proteins, lacking their *N*-terminal transmembrane segments. The genetic sequence of soluble PBP3 from *P. aeruginosa* (*Pa*PBP3ΔTM, res. 40-563) was cloned into pET41b(+) at restriction sites NdeI/HindIII. A thrombin cleavage site was introduced using restriction sites HindIII/NotI to facilitate removal of the *C*-terminal 8xHis-tag. The coding sequence of soluble PBP3 from *E. coli* (*Ec*PBP3ΔTM, res. 49-588) was cloned by NdeI/BamHI into pET28a(+) vector, resulting in a thrombin-cleavable 6xHis-tag at the *N*-terminus. A cDNA coding for the transpeptidase domain of *E. coli* PBP3 (*Ec*TPd*, S68-V88–G_3_–E164-Q203–G_3_–A228-T570) with *N*-terminally fused 6xHis-tag and TEV cleavage site sequences was obtained as a synthetic gene (Biomatik) and inserted into pET28a(+) at NcoI/BamHI restriction sites. Expression of all constructs was performed in *E. coli* BL21(DE3) (Novagen). At OD_600_ = 0.5, protein expression was induced with 1 mM IPTG for 20 h at 20 °C. Cells were harvested by centrifugation, washed and pellets stored at −80 °C. Cells were enzymatically lysed by QProteome Lysis Kit (Qiagen) using 25 mL lysis buffer per liter of culture volume according to the manufacturer’s instructions. After centrifugation for 1 h at 27,000× *g* and 4 °C, soluble His-tagged proteins were purified by Ni^2+^-NTA affinity chromatography. *Pa*PBP3ΔTM bound to Ni^2+^-NTA resin was washed with BufferW*Pa* (10 mM Tris-HCl, 200 mM NaCl, 30 mM imidazole, pH 7.5) and eluted by on-column thrombin cleavage for 16 h at 20 °C in BufferW*Pa*. *Ec*PBP3ΔTM was treated the same way but using BufferW*Ec* (15 mM KH_2_PO_4_, 2 mM Na_2_HPO_4_, 200 mM NaCl, 30 mM imidazole, pH 7.5). *Ec*TPd* bound to Ni^2+^-NTA resin was washed with BufferW*Ec* and eluted by imidazole with E300 buffer (15 mM KH_2_PO_4_, 2 mM Na_2_HPO_4_, 100 mM NaCl, 300 mM imidazole, pH 7.5). The 6xHis-tag of *Ec*TPd* was removed by TEV-protease cleavage for 16 h at 20 °C. In a final step, all proteins were purified by size exclusion chromatography using a HiLoad^TM^ 16/600 Superdex 75 pg column (GE Healthcare, Cytiva). Monomeric peak fractions were analyzed by SDS-PAGE, pooled, dialyzed, and concentrated. *Ec*PBP3ΔTM and *Ec*TPd* were stored at concentrations of approx. 10 mg/mL at 4 °C in 15 mM KH_2_PO_4_, 2 mM Na_2_HPO_4_, 100 mM NaCl, pH 6.0 for several months. *Pa*PBP3ΔTM was stored at concentrations of approx. 2 mg/mL at 20 °C in 10 mM Tris-HCl, 200 mM NaCl, 20% (*v*/*v*) glycerol, pH 7.5 for several weeks.

*Crystallization.* All crystallization experiments were set up at 293 K using the sitting drop approach where 0.5 µL of each precipitant solution was mixed with 0.5 µL of protein solution. Crystals of *Ec*PBP3ΔTM:AIC499 were observed in 3% (*w*/*v*) dextran sulfate (M-5000), 0.1 M sodium cacodylate, 5% (*w*/*v*) PEG 8000 and 30% (*v*/*v*) MPD at pH 6.5 with a protein concentration of 10 mg/mL in the presence of 500 µM AIC499 after a few days. Crystals of *Ec*TPd* were observed in 0.1 M sodium cacodylate, 5% (*w*/*v*) PEG 8000, 20% (*v*/*v*) MPD, 0.2% (*w*/*v*) betaine, 0.2% (*w*/*v*) L-glutamic acid, 0.2% (*w*/*v*) L-proline, 0.2% (*w*/*v*) taurine, 0.2% (*w*/*v*) trimethlyamine N-oxide, and 0.02 M HEPES at pH 6.5 with a protein concentration of 14.4 mg/mL after a few days. The AIC499 complex was crystallized in 0.1 M MES and 25% (*w*/*v*) PEG 1000 at pH 6.5 with a protein concentration of 10 mg/mL in the presence of 500 µM AIC499 after one week. The structures derived from *Ec*PBP3 were determined by molecular replacement using an appropriately modified version of the published *Ec*PBP3ΔTM structure (PDB entry 4BJP [13]). *Pa*PBP3ΔTM crystals were observed in 0.1 M Na_2_SO_4_ and 24% (*w*/*v*) polyvinylpyrrolidone with a protein concentration of 8 mg/mL (apo crystal form 1). In the presence of 500 µM AIC499, and with a protein concentration of 14 mg/mL, *Pa*PBP3ΔTM:AIC499 crystals formed in 20% (*v*/*v*) Jeffamine^®^ M-2070 and 20% (*v*/*v*) DMSO and were cryoprotected by adding 1 µL glycerol to the crystallization drop. In co-crystallization experiments with a different PBP3-targeting compound, two additional *Pa*PBP3ΔTM crystal forms were observed after four to six weeks in (i) 0.1 M MES, 5% (*w*/*v*) PEG 3000, 30% (*v*/*v*) PEG 200, pH 6.0, and (ii) 0.2 M potassium/sodium tartrate, 20% (*w*/*v*) PEG 3350, using 9 mg/mL *Pa*PBP3ΔTM, and 500 µM of the compound dissolved in DMSO. Because the respective structures were found not to contain the compound, they are treated as *de-facto* apo structures, and these crystalline states are hence designated apo crystal forms 2 and 3 in this work. For determining the *Pa*PBP3ΔTM and *Pa*PBP3ΔTM:AIC499 structures, a model derived from PDB entry 3OC2 was used in molecular replacement [25].

Prior to measurements, all crystals were flash-cooled to 100 K and stored in liquid nitrogen. Diffraction data were collected at the European Synchrotron Radiation Facility (ESRF; Grenoble, France) on beam line ID23-1 and at the Deutsches Elektronen-Synchrotron (DESY; Hamburg, Germany) on beam lines P11 and P13 (operated by EMBL). Raw data were processed with XDS, followed by anisotropic truncation in STARANISO [26,27] using default settings. Following molecular replacement using MOLREP [28] (refer to Results section for choice of search structures), models were iteratively improved by reciprocal-space refinement in phenix.refine [29], including TLS (translation, libration, screw) parameterization, with grouping suggested on the basis of refined B-factors, and manual rebuilding using COOT [30]. Data collection and refinement statistics are provided in Table 1. Figures were prepared using PyMol and LigPlot+ [31,32]. LigPlot+ graphs in Figures 4 and 6 were created using the superposition option to ensure consistent orientation, and thresholds for plotting non-covalent interactions were modified as indicated in the respective legends.

*NMR experiments.* Uniformly [^2^H, ^13^C, ^15^N]-labeled *Ec*PBP3ΔTM and *Ec*TPd* were expressed following the protocol from Cai et al. [33] and purified as described above. *Ec*PBP3ΔTM (111 µM) and *Ec*TPd* (60 µM) NMR samples were prepared in Sörensen buffer (15 mM KH_2_PO_4_, 2 mM Na_2_HPO_4_, 100 mM NaCl, pH 6.0) and 1 mM 4,4-dimethyl-4-silapentanesulfonic acid (DSS) with 5% (*v*/*v*) D_2_O. NMR spectra were recorded at 37 °C on a Bruker Avance III HD NMR spectrometer equipped with a TCI cryoprobe operating at a ^1^H frequency of 700 MHz. The temperature was calibrated using predeuterated methanol [34]. Data were processed via NMRPipe and analyzed using CcpNMR analysis [35,36].

*Nanoscale differential scanning fluorometry.* Protein thermal stability was determined by differential scanning fluorometry (DSF) using a Prometheus Panta device (NanoTemper Technologies). There was 20 µM protein mixed with 500 µM aztreonam or AIC499, or 5% DMSO as a control, in appropriate buffers (*Pa*PBP3ΔTM: 10 mM Tris-HCl, 200 mM NaCl, 20% (*v*/*v*) glycerol, 0.01% (*v*/*v*) Triton X-100, pH 7.5; *Ec*PBP3ΔTM: 15 mM KH_2_PO_4_, 2 mM Na_2_HPO_4_, 200 mM NaCl, 20% (*v*/*v*) glycerol, 0.01% (*v*/*v*) Triton X-100, pH 7.5). Samples were centrifuged for 10 min at 19,000× *g* and 4 °C. Standard capillaries were filled with 10 µL samples, and temperature was increased from 20 °C to 80 °C at a heating rate of 1 °C/min. Fluorescence at 330 and 350 nm was continuously measured, and ratios and first derivatives were calculated using the software provided by the manufacturer (PR. Panta Control v1.0.7/PR. Panta Analysis v1.0.1).

## 3. Results

### 3.1. Structures of E. coli PBP3

#### 3.1.1. *E. coli* PBP3 Apo Protein

To simplify handling, PBP3 from *E. coli* was produced as a soluble version in which the *N*-terminal 48 residues, including the transmembrane helix, were removed (*Ec*PBP3ΔTM).

**Figure 2:**
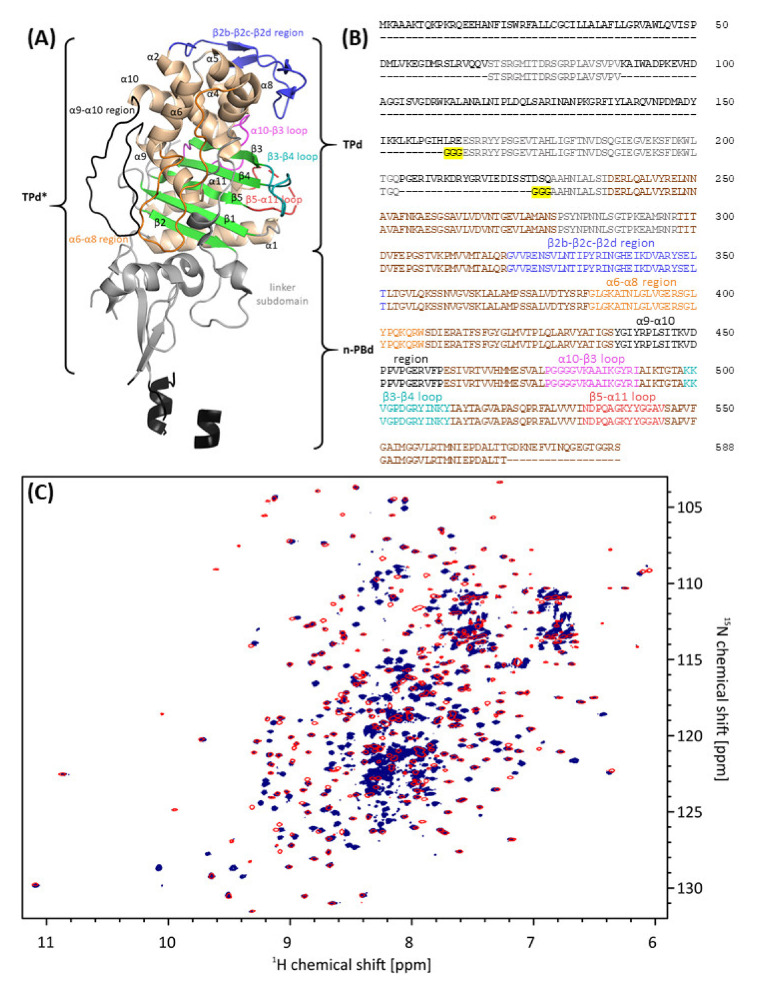
Design of an *Ec*TPd* construct for structural studies. (**A**) X-ray structure of *Ec*PBP3ΔTM published previously (PDB entry 4BJP, [13]). Secondary structure elements and intervening loops relevant to this paper are labeled. Note that the n-PBd of the elongated molecule has been traced only incompletely. (**B**) Sequence alignment of *Ec*PBP3ΔTM (upper sequence) and the newly designed *Ec*TPd* construct (bottom sequence) performed with Clustal Omega [41]. The structural elements labeled in (**A**) as well as the tri-glycine linkers (yellow background) are highlighted in the sequence. (**C**) Overlay of 2D ^1^H-^15^N TROSY-HSQC NMR spectra of uniformly [^2^H, ^13^C, ^15^N]-labeled *Ec*PBP3ΔTM (blue) and *Ec*TPd* (red) demonstrates the structural similarity of both proteins in solution.

Apo-*Ec*PBP3ΔTM was readily crystallized, but crystals yielded weak and anisotropic diffraction data extending to a maximum resolution of 4 Å, which prevented determination of its three-dimensional structure. We reasoned that the *N*-terminal part of the protein comprising the n-PBd may prevent formation of a highly ordered crystal lattice because of its flexibility. This view is supported by a crystal structure published previously (PDB entry 4BJP) that features very high B-factors and partial absence of electron density, particularly in the head and anchor subdomains (nomenclature according to [37]; also refer to Figure 5). For this reason, a truncated version of *Ec*PBP3ΔTM was designed that included the catalytical TPd and had certain segments replaced with tri-glycine linkers. The resulting construct (S68-V88–G_3_–E164-Q203–G_3_–A228-T570; *Ec*TPd*) is presented in Figure 2B. In contrast to a previously published *Ec*TPd structure (PDB entry 6HZQ [38]), the truncation was defined strictly based on tertiary fold, ensuring that regions S68-V88 and E164-Q203 of the closely apposed linker subdomain (colored grey in Figure 2) were included.

Acquisition of two-dimensional (2D) ^1^H^-15^N transverse relaxation-optimized spectroscopy (TROSY) heteronuclear single quantum correlation (HSQC) NMR spectra of uniformly [^2^H, ^13^C, ^15^N]-labeled *Ec*PBP3ΔTM and *Ec*TPd* revealed that most peaks in the 2D ^1^H^-15^N TROSY-HSQC spectrum of *Ec*TPd* matched peaks in the corresponding spectrum of *Ec*PBP3ΔTM (Figure 2), indicating that the transpeptidase and linker domains in the two proteins adopt a similar three-dimensional fold [39]. Many peaks in the spectrum of *Ec*PBP3ΔTM that are absent in the one of *Ec*TPd* have random coil ^1^H chemical shifts (between 8.00 and 8.43 ppm [40]), indicating that regions in the head and anchor subdomains are highly dynamic. This observation is consistent with our hypothesis, based on the initial crystallographic efforts and literature described above, that the n-PBd of *Ec*PBP3ΔTM displays enhanced flexibility. As expected, the average line width of the *Ec*TPd* peaks narrowed because of a decrease in the rotational correlation time, and variation in intensities among peaks decreased by removing the more dynamic regions of *Ec*PBP3ΔTM. The good spectral dispersion of peaks in the 2D ^1^H-^15^N TROSY-HSQC of *Ec*TPd* indicates that structure and dynamic studies of this protein by NMR are feasible.

After showing that the solution structure of the *Ec*PBP3 TPd is largely unaffected by the truncation, the *Ec*TPd* protein was crystallized. Indeed, these crystals were found to exhibit considerably improved diffraction quality (including reduced anisotropy) when compared with those obtained using *Ec*PBP3ΔTM, with useful data extending to a resolution of approx. 2.3 Å. The final model, featuring hexagonal space group P 6_2_ 2 2 with one molecule per asymmetric unit, contains residues T69-T569 of *Ec*TPd* (Table 1).

**Figure 3:**
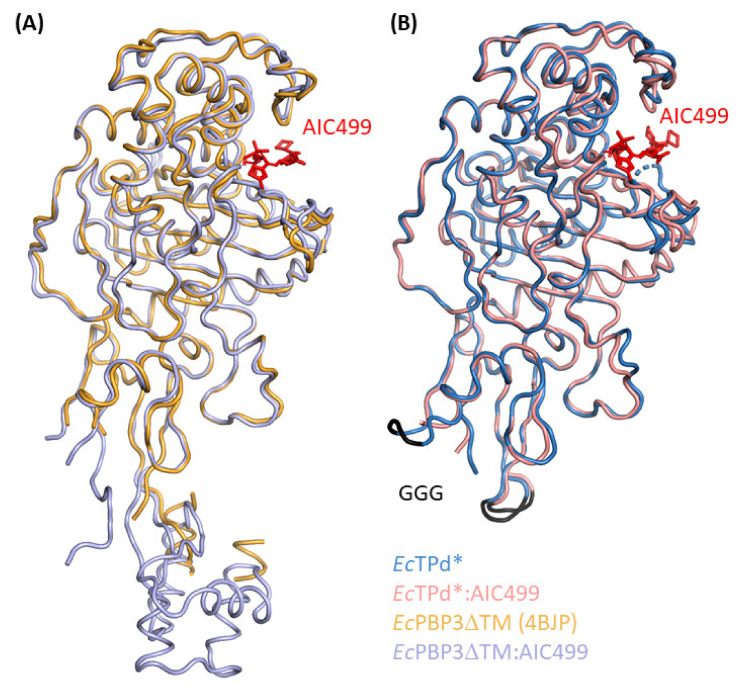
X-ray structures of *Ec*PBP3 determined in the absence and presence of AIC499 (red stick model). (**A**) The published apo-*Ec*PBP3ΔTM structure (PDB entry 4BJP [13], gold) is used for superposition with the AIC499-complexed *Ec*PBP3ΔTM (blue, this study). (**B**) The structure of apo-*Ec*TPd* is shown in dark blue, while the complex with AIC499 is colored salmon. The GGG linkers replacing the removed segments are shown in black.

Comparison of the *Ec*TPd* structure (Figure 3B, dark blue) with the *Ec*PBP3ΔTM structure published previously (PDB entry 4BJP [13]; Figure 3A, gold) confirms that the 3D fold is mostly identical; 324 common C_α_ atoms superimpose with an overall root-mean-square (RMS) distance of 0.39 Å. As expected, notable differences are observed close to the truncation sites, i.e., the V88–G_3_–E164 and Q203–G_3_–A228 regions, which are in contact with the β5-α11 and α9-α10 segments, respectively, of symmetry equivalent molecules (designation of PBP3 secondary structure elements according to [37]). These lattice interactions, in turn, cause the α9-α10 backbone to shift outwards by up to 2 Å; the β5-α11 region has not been traced in the *Ec*PBP3ΔTM structure, but electron density indicates a relative displacement on the order of 5 Å. Additional differences in the vicinity of the catalytic center concern the β2b-β2c-β2d region as well as the β3-β4 hairpin. While the former is probably affected by a lattice contact, chiefly mediated by the adjacent α5-α6 linker, and the segment preceding helix α8, contacting their respective equivalents in a symmetry mate, the latter appears to be similarly influenced by the cognate region in a neighboring copy, with distances suggesting a repulsive electrostatic interaction. Since all of these moderate alterations are far from the truncation sites, and can be readily explained by differences in lattice contacts, we conclude that the structure of *Ec*TPd* is largely representative of the *Ec*PBP3ΔTM protein, in agreement with our observations using NMR spectroscopy.

#### 3.1.2. Effect of AIC499 Binding on *E. coli* PBP3

**Figure 4:**
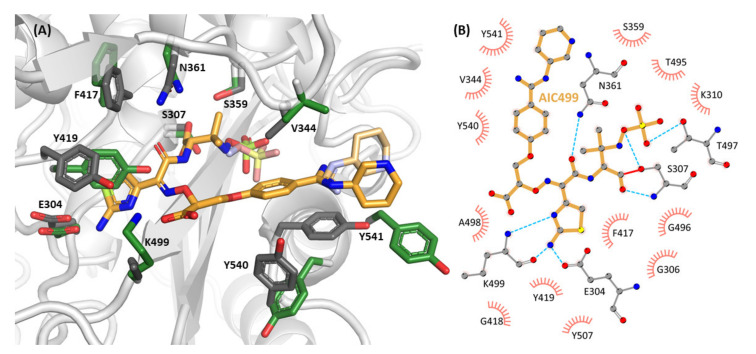
Structure of AIC499 within the active site of *Ec*TPd*. (**A**) 3D representation of the covalently bound ligand together with the most relevant interacting side chains (gray) and their counterparts in the apo structure (green). The second conformation of the amidine-based head group, as well as the terminal sulfate moiety are shown in lighter color. Note that K499 has been truncated in the complex structure because of missing electron density. (**B**) LigPlot+ representation of individual contacts for one conformer. Hydrogen bonds (cyan) are plotted for donor-acceptor distances between 2.3 Å and 3.2 Å, while hydrophobic interactions (salmon) have distances between 3.0 Å and 4.0 Å. A complete list of distances between protein side chains and the AIC499 compound is provided in Table A1.

The *Ec*PBP3ΔTM and *Ec*TPd* proteins were crystallized after pre-incubation with the experimental β-lactam AIC499. In the case of the truncated version, the presence of the compound left the space group (P 6_2_ 2 2) and packing unchanged. It did, however, impart a notable improvement in useable resolution, along with slight changes in lattice constants. For the *Ec*PBP3ΔTM:AIC499 complex, we observed space group P 6_4_ 2 2, which differs from the symmetry reported previously for the apo protein (PDB entry 4BJP [13], space group P 6_1_ 2 2); again, diffraction quality was clearly improved by the presence of the ligand but was still inferior to *Ec*TPd*:AIC499 (Table 1). To understand the effects of structural optimization leading to the final AIC499 compound, the environment of the five functional groups addressed during the process (Figure 1) was analyzed in the crystal structures of *Ec*TPd* and *Ec*PBP3ΔTM complexes. Since there were only negligible differences between the two models (RMS distance: 0.37 Å for 336 equivalent C_α_ positions), we will focus on the binding of AIC499 to *Ec*TPd* (Figure 3B and Figure 4) because (i) electron density was clearly more informative in this structure, and (ii) despite differences in crystallization conditions, the complex has been crystallized in the same space group as the apo form, minimizing spurious differences caused by non-conserved lattice contacts.

As expected for a β-lactam antibiotic, AIC499 is found as a covalent acyl-enzyme intermediate, with the carbonyl group of the hydrolyzed lactam forming an ester with the side chain oxygen of the catalytic S307. The compound adopts a U-shaped overall conformation, and its presence correlates with several conformational changes in the protein environment, mostly regarding the β2b-β2c-β2d, β3-β4, and β5-α11 regions, which contribute to the upper lobe, the bottom, and the lower lobe, respectively, of the binding cleft.

The largest differences between apo and complex structures can be observed in the β5-α11 region, in particular residues K539-A544. Being poorly ordered and not completely resolved in the native protein, this segment is well-structured when AIC499 is bound in the active site. Indeed, ordering of the β5-α11 loop has been observed regularly as a result of PBP-β-lactam interaction [25]. Residues Y540 and Y541 appear to be particularly relevant here, because they re-orient towards the ligand and, together with Y511 from strand β4, shield the binding pocket for the R^1^ substituent of the compound as an “aromatic wall”. These hydrophobic interactions centered on the phenyl ring of the head group are accompanied by a notable reduction in B-factors in the side chains involved.

Strands β3 and β4 are slightly shifted towards the core of the domain, relative to the remainder of the central β-sheet, with the protruding twisted hairpin being displaced in the opposite direction. Interactions between residues in the β3 strand and AIC499 include coordination of the terminal sulfate moiety (R^2^) by the T497 side chain, rotation of the T495-G496 peptide plane (belonging to the third catalytic motif, KS/TG, of the canonical penicillin binding domain) because of steric interference, and formation of a hydrogen bond between the K499 carbonyl and the primary amine of the amino-thiazole group (R^4^). A less favorable bond is potentially formed between the K499 amide nitrogen and either of the two amines in group R^4^. Interestingly, the K499 side chain does not interact with the carboxyl group of the AIC499 linker region even though the apo structure suggests this side chain is in a favorable position for such an interaction. In fact, the side chain becomes less ordered in the presence of the compound, possibly alternating between various hydrogen bonding partners, which prohibits accurate modeling. The impact of AIC499 on strand β4 is mediated mostly by hydrophobic contacts. In addition to Y511 mentioned above, side chains of Y507 and Y514 are both involved in aromatic clusters. Y507 apparently moves in concert with Y419 in the proximal α8-α9 loop, which is displaced by and aligns parallel to the AIC499 amino-thiazole moiety, forming a π-π stack. Y514, on the other hand, propagates its displacement by the β3 strand on to F303 in the β2-α2 linker; the flipped side chain of F303 engages in a hydrophobic cluster with I512 and the aliphatic portion of K500, thus linking back to the β3-β4 region. Remarkably, while the side chain of S307 is involved in the acyl-enzyme intermediate, and E304 establishes an important hydrogen bond with the amino-thiazole moiety, the main chain of the 300s region is only moderately affected by compound binding. Together with helix α8, the whole upper lobe comprising the β2b-β2c-β2d region and the adjacent helices α4 and α5 moves slightly towards the active site, allowing some side chains to interact with AIC499. In particular, V344 between strands β2c and β2d appears to play an important role in stabilizing AIC499 by hydrophobic interactions. On the one hand, it is in Van der Waals (vdW) contact to the phenyl ring of the amidine group (R^1^), thus forming the counterpart of the aromatic wall on the opposite face of the compound. On the other hand, it also favorably interacts with one of the methyl groups at the C-4 position of the former β-lactam ring. Furthermore, the side chains of S359 and N361 (part of the second catalytic motif, SXN) preceding helix α5 are hydrogen-bonded to the acyl-enzyme ester oxygen and carboxylamide oxygen atoms, respectively, of AIC499.

The piperidine moiety of the head group R^1^ does not engage in strong interactions with the protein and is modeled in two alternate conformations, with one edge of the ring in vdW contact with the Y541 side chain. Thus, this part of AIC499 does not seem to be very important for stabilizing the bound state.

We note certain structural differences between apo-*Ec*TPd* and *Ec*TPd*:AIC499 in regions distant from the active center of *Ec*PBP3. In addition to the truncation sites, this also concerns neighboring segments with enhanced flexibility, specifically the *N*-terminus, the β5n-β6n loop, and the vicinity of helix α1b. These alterations can be explained by the extensive conformational change in the β5-α11 region, which is in direct contact with the V88–G_3_–E164 hairpin of a symmetry-equivalent copy, together with differences in crystallization conditions, resulting in the presence of a PEG molecule linking the V88–G_3_–E164 region to the *N*-terminal segment in the complex but not in the apo structure. The region preceding and including helix α1b is remarkable because it shifts more than 2 Å away from its conformation in the apo structure, which exceeds the differences in its immediate neighborhood, without itself being involved in a lattice contact. We speculate that these differences are an additive result of long-range conformational triggers propagated from the ligand binding site on the one hand and the “truncation pole” on the other.

### 3.2. Structures of P. aeruginosa PBP3

#### 3.2.1. *P. aeruginosa* PBP3 Apo Protein

As outlined above for *E. coli* PBP3, a soluble version of the *P. aeruginosa* orthologue was produced lacking the *N*-terminal transmembrane helix (*Pa*PBP3ΔTM). Remarkably, three distinct crystal forms were identified for *Pa*PBP3ΔTM, featuring different unit cells and lattice contacts but all belonging to space group C 1 2 1. Diffraction quality was generally superior when compared to *Ec*PBP3ΔTM crystals, with moderate anisotropy and usable resolutions of 2.2 Å (crystal form 1), 1.8 Å (crystal form 2), and 1.9 Å (crystal form 3; for details refer to Table 1).

**Figure 5:**
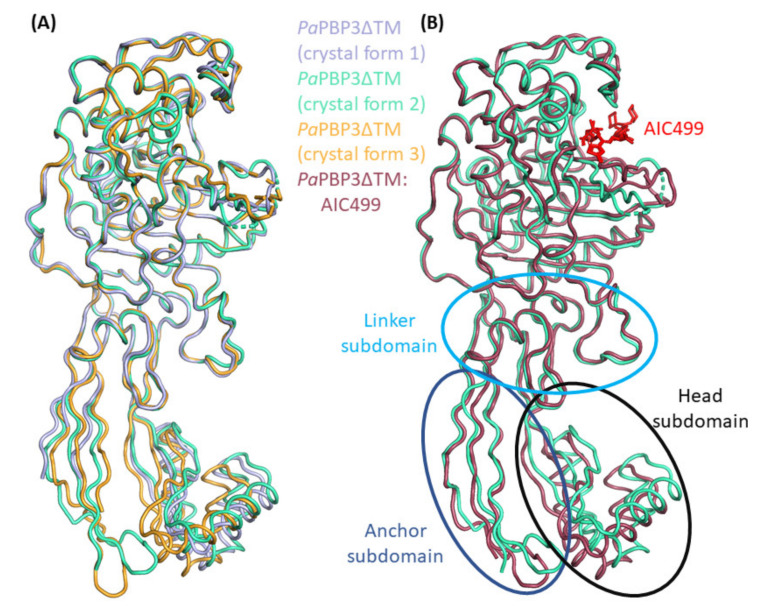
X-ray structures of *Pa*PBP3 determined in the absence and presence of AIC499 (red stick model). (**A**) Crystal form 1 (blue), crystal form 2 (green) and crystal form 3 (bright orange) of *Pa*PBP3ΔTM feature different unit cells, leading to slightly different orientations predominantly in the n-PBd. (**B**) The structure of apo-*Pa*PBP3ΔTM crystal form 2 (cyan) is used for superposition with *Pa*PBP3ΔTM:AIC499 chain B (dark red). Additionally, the head, anchor, and linker subdomains of the n-PBd are highlighted with black, dark blue, and light blue ellipses, respectively.

As expected, superposition of the three models reveals a very similar overall structure (Figure 5A). The RMS distance between corresponding C_α_ positions was calculated between 0.50 Å and 0.65 Å, but values decrease to approx. 0.30 Å if only the TPase fold (R62-S76, R152-A187, and K217-A563) is considered. In fact, the head subdomain (T77-R152) appears to largely bend as a rigid body, leading to slightly different orientations. These can be explained by packing effects; in addition to a lattice contact shared by all three structures (α1n-α2n region and α3n helix with the α2-β2a loop and the proximal α10 helix of a symmetry mate), crystal forms 1 and 3 feature unique interactions involving, among others, the α3n helix and β3n-α4n regions, respectively, contacting the β2e-β2f and α2-β2a segments, or the β2b-β2c hairpin, of neighboring molecules.

The anchor subdomain is non-contiguous, consisting of the *N*-terminal segment (A50-H61) and a long β-hairpin (G188-P215), and despite its spatial proximity moves independently of the head domain. This domain features particularly high flexibility, as evidenced by large B-factors and the difficulties of consistent tracing. In fact, the final models for crystal forms 2 and 3 lack several side chains in this region as well as small portions of the extreme *N*-terminus because of missing electron density, whereas in crystal form 1, larger parts of the backbone could not be traced. Again, the differences can be explained by packing effects.

In the TPd, the largest differences between the three apo-*Pa*PBP3ΔTM structures concern the β3-β4 and β5-α11 regions, both of which show indications of high flexibility. The β3-β4 hairpin is generally non-contiguous in electron density, preventing it from being modeled completely. In crystal forms 1 and 2, eight and six residues are not resolved, respectively, while in crystal form 3 nine residues are missing. In contrast to the structure in crystal forms 1 and 2, the protruding part of the β3-β4 region of crystal form 3 seems to bend towards the active site. This difference correlates with a lattice contact established with helix α1 that is exclusive to form 3. Based on previously solved apo-*Pa*PBP3ΔTM structures (PDB entries 3OC2, 3PBN and 6HZR), the β3-β4 hairpin was thought to be disordered and to be stabilized upon a ligand (or substrate) binding to the active site [11,25,38]. Our observations indicate that the protruding β3-β4 segment is at least partly structured in the apo protein, even if not engaged in lattice contacts.

While it was not possible to completely model the β3-β4 loop in any of the three crystal forms, we were able to trace the β5-α11 region at least in crystal form 1. In contrast, two and five residues, respectively, are missing in the other two forms. This observation might be related to a packing effect caused by the head subdomain of a neighbor molecule restricting mobility of the β5-α11 region in form 1 (indirectly via helix α1) to a bent conformation. In contrast, this segment appears to adopt a more extended structure in crystal form 2, as judged by the resolved portion. Comparison with published *Pa*PBP3ΔTM apo structures confirms the notion of enhanced conformational freedom of the β5-α11 loop. While it is bent towards the active site in crystal form 1, it is orientated in the opposite direction in the 6HZR structure and is essentially absent in 3OC2. The 3PBN structure adopts what may be considered an intermediate state, since the β5-α11 loop is not bent strongly in either direction; however, this is, again, the result of a lattice contact with a neighboring head subdomain.

#### 3.2.2. Effect of AIC499 Binding on *P. aeruginosa* PBP3

In addition to the apo structures, *Pa*PBP3ΔTM was produced and crystallized in the presence of AIC499; crystals belonged to space group P 2_1_ 2_1_ 2_1_ with two copies per asymmetric unit and yielded diffraction data extending to a resolution of 1.7 Å (Table 1). As expected, the AIC499 complex structure displays high overall similarity to the apo version, with overall RMS distances (chain B) of 0.55 Å, 0.58 Å, and 0.57 Å w.r.t. crystal forms 1, 2, and 3, respectively. The two molecules in the asymmetric unit differ from each other and from the apo structures in the orientation of the head and anchor subdomains relative to the TPd. Similar to the variation among the apo-*Pa*PBP3ΔTM structures, this effect can be explained by slight bending of β-strands dominating these extended folds, induced by different lattice environments. Specifically, in addition to the highly favorable contact described above for all three apo structures, chain A displays additional extensive interactions of its *N*-terminal part (strands β1n and β9n, α4n-β4n loop) with helix α5n in the linker subdomain, the α6-β2e loop and helix α1 of symmetry-related copies, while in chain B a contact of strand β9n with the β2h-β2i segment of a symmetry mate is noteworthy. Within the asymmetric unit, the extended *C*-terminus of chain A is in contact with the head and linker subdomains of chain B; the reciprocal interaction is not observed.

As described above for the complex with the *E. coli* protein, the AIC499 molecule is covalently associated with *Pa*PBP3ΔTM via the catalytic serine (S294) side chain. In the active site environment, the β3-β4 loop as well as the β5-α11 loop appear quite flexible, despite the presence of the ligand, as evidenced by high B-factors and often discontinuous electron density. Nevertheless, we note that strands β3 and β4 clearly bend towards the active site, supporting some interactions of their side chains with the AIC499 molecule, most importantly R489 (see below). In contrast, the β5-α11 loop is ordered in chain B only and bends in a similar direction to that observed in apo form 2. More importantly, helix α11 is *N*-terminally extended by more than one turn in both chains, which again allows additional contacts with the ligand.

**Figure 6:**
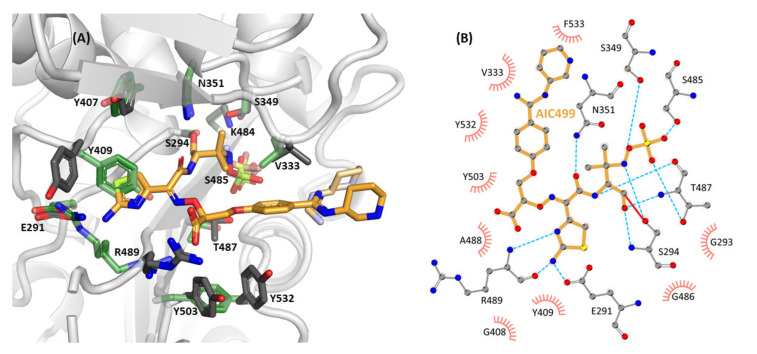
Structure of AIC499 within the active site of *Pa*PBP3ΔTM. (**A**) 3D representation of the covalently bound ligand together with the most relevant interacting side chains (gray) and their counterparts in the apo structure (crystal form 2, green). Alternative conformations of the amidine-based head group and the terminal sulfate moiety are shown in lighter color. (**B**) LigPlot+ representation of individual contacts. Hydrogen bonds (cyan) are plotted for donor-acceptor distances between 2.3 Å and 3.2 Å, while hydrophobic interactions (salmon) have distances between 3.0 Å and 4.0 Å. A complete list of distances between protein side chains and the AIC499 compound is provided in Table A1.

The overall conformation and interactions of AIC499 in its complex with *Pa*PBP3ΔTM (Figure 6) resemble those described above for the *Ec*PBP3ΔTM adduct. The terminal sulfate group (R^2^) is hydrogen bonded to the side chains of K484, S485, and T487 (the region of the third signature motif). The nitrogen of the former β-lactam ring forms a hydrogen bond with the hydroxyl moiety of S349, whereas the ligand amide group interacts with the carbonyl of T487 on the one hand and with the side chain amide of N351 on the other.

The amino-thiazole (R^4^) is well stabilized, engaging in three hydrogen bonds with the side chain of E291 (specifically, one of its alternate conformations) and the backbone of R489. Unlike the situation in the *Ec*PBP3ΔTM:AIC499 complex, the amino-thiazole does not form a parallel π-π stack with Y409 (equivalent to Y419 in *Ec*PBP3) but a displaced T-stack. Additionally, Y407 (equivalent to F417 in *Ec*PBP3) is close to the aromatic rings of Y409 and the amino-thiazole, establishing an aromatic network for further stabilization.

The carboxyl group of the linker (R^5^) forms hydrogen bonds to the guanidinium group of R489, and the phenyl ring of the head group (R^1^) is close to the aliphatic side chain of V333. On the opposite face of this ring, another aromatic network is formed: the R^1^ phenyl ring stacks with Y532, which is further stabilized by parallel π-π stacking with Y503. Additionally, C_β_ of F533 appears close to the aromatic ring of AIC499, but the electron density was too weak to build the remainder of the side chain. Interestingly, with an occupancy of 40%, it was possible to model an alternative set of correlated side chain conformations for R489 and Y503, located in the β3 and β4 strand, respectively. Due to a clash of the alternate Y503 rotamer with Y532, the β5-α11 loop must be displaced as well; however, electron density was not conclusive as to the respective conformer.

Similar to the complex with *Ec*PBP3ΔTM, the AIC499 head group (R^1^) appears with two conformations in both copies present in the asymmetric unit. In general, the piperidine moiety is devoid of strong interactions with the protein; while in chain B V333 at a vdW distance from either variant is the only notable contact, one of the conformers in chain A orients towards Y532.

## 4. Discussion

The novel monobactam AIC499 was developed as a drug candidate to address a major shortcoming in today’s antimicrobial toolkit, i.e., decreasing efficiency because of acquired multi-drug resistance of pathogenic bacterial strains. In this work, we have investigated the structural foundations of AIC499 activity using PBP3 from Gram-negative bacteria *E. coli* and *P. aeruginosa* as model targets. The crystal structures described herein feature the long-lived acyl-enzyme intermediates formed with the compound and thus capture the end point of mechanism-based (“suicide”) inhibition. It should be noted that the actual acylation reaction is preceded by formation of a non-covalent Michaelis complex, and the structural properties of the latter may differ to some extent from those of the final covalent adduct. In the crystal structures, including AIC499, however, the covalent linkage appears essentially “neutral” with respect to the non-covalent interactions of the compound, suggesting that distortions related to acylation are probably small.

The crystal structures of PBP3, in complex with AIC499, reveal that the extent of non-covalent interactions with AIC499 differs slightly between the *P. aeruginosa* and *E. coli* proteins (Figure 4 and Figure 6). For example, a total of eleven hydrogen bonds are formed with *Pa*PBP3, whereas only seven are formed in the *Ec*PBP3 complex, which may reflect that *Pa*PBP3 was used as the target during optimization. In both cases, the amino-thiazole (R^4^) is the functional group exhibiting the highest density of interactions with PBP3. Besides the hydrogen bonds established with residues E291 and R489 (E304 and K499 in *Ec*PBP3) by the nitrogen-containing half of the heterocycle, the less polar moiety engages in an aromatic network with the side chains of Y409 and Y407 (Y419 and F417). In a similar fashion, the phenyl ring of the head group (R^1^) is in intimate contact with both proteins, being sandwiched between an aromatic cluster (Y511, Y540, Y541 in *Ec*PBP3; Y503, Y532, F533 in *Pa*PBP3) and the aliphatic V344/V333 side chain. The piperidine ring of the head group, on the other hand, seems to have relatively low impact on the binding of AIC499; in fact, it appears to be the most dynamic part with two discernible conformations in either complex. In contrast to Y541 in *Ec*PBP3, the homologous F533 in *Pa*PBP3 is disordered, and the piperidine moiety instead contacts Y532 or V333. While this modification was found to improve the pharmacodynamic and pharmacokinetic properties of AIC499 compared to its predecessors (data not shown), its benefit is thus not immediately obvious from the X-ray structures alone. We hypothesize that the piperidine substituent may instead impact the kinetics or thermodynamics of the primordial Michaelis complex; clarification of this issue will require further experimentation, applying catalytically inactive PBP3 variants. Notable differences between *E. coli* and *P. aeruginosa* complexes are observed for the carboxyl moiety of the linker (R^5^); in *Pa*PBP3 the side chain of R489 forms a salt bridge with this group, whereas the homologous K499 in *Ec*PBP3 is partly disordered. This is consistent with the notion that arginine is more versatile in establishing electrostatic interactions because of its geometric properties [42].

**Figure 7:**
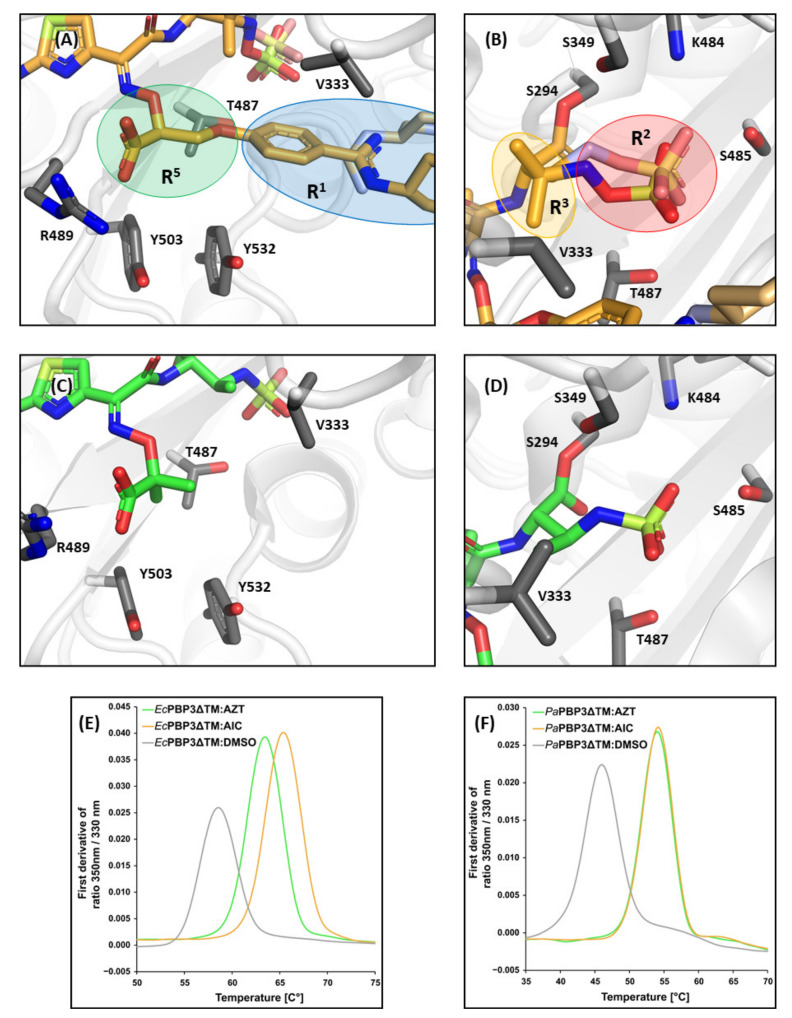
Comparison of aztreonam and AIC499 binding to *Pa*PBP3 and *Ec*PBP3. Panel (**A**) shows a close-up view of the linker (R^5^; green) and head group (R^1^; blue) of AIC499 in complex with *Pa*PBP3ΔTM, while (**B**) focuses on the sulfate (R^2^; red) and dimethyl group (R^3^; orange). The analogous views of aztreonam in complex with *Pa*PBP3ΔTM (PDB entry 3PBS [11]) are represented in (**C**) and (**D**), respectively. Refer to Table A2 for a complete list of distances between protein side chains and the aztreonam molecule. (**E**) DSF data obtained with *Ec*PBP3ΔTM; the first derivative of the 350 nm/330 nm fluorescence ratio is plotted against the temperature. The control experiment with DMSO added to the protein (gray) yields a *T*_m_ of 58.6 °C, which increases to 63.4 and 65.4 °C after addition of 500 µM aztreonam (AZT, green) and AIC499 (AIC, gold), respectively. (**F**) Analogous experiments with *Pa*PBP3ΔTM measured in complex with aztreonam (green) and AIC499 (gold) gave nearly identical *T*_m_ values of 54.0 and 54.1 °C, respectively. The *T*_m_ for the control experiment (gray) was determined to be 46.0 °C. Raw data of the thermal shift experiments are shown in Figure A1.

**Figure A1:**
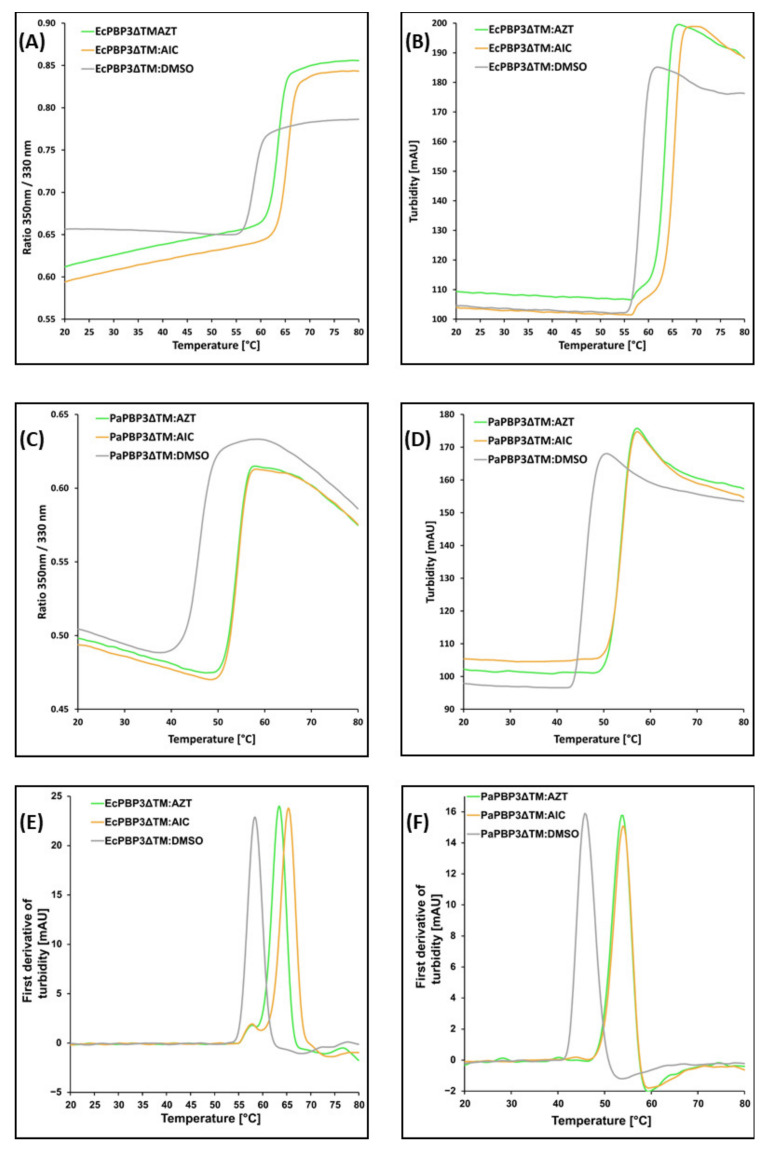
Raw data of the thermal shift experiments. In the upper row, the ratio of 350 nm/330 nm fluorescence (**A**), the turbidity (**B**) are plotted against temperature. *T*_m_ derived from turbidity measurements were determined to be 58.4 °C for apo-*Ec*PBP3ΔTM, and 63.4 and 65.3 °C for *Ec*PBP3ΔTM in complex with aztreonam and AIC499, respectively. In the middle row, the analogous data are shown for *Pa*PBP3ΔTM (**C**,**D**). *T*_m_ were calculated to be 45.9, 53.8 and 54.0 °C for the DMSO control, *Pa*PBP3ΔTM in complex with aztreonam and with AIC499, respectively. In the bottom row, the first derivatives of the turbidity are plotted for *Ec*PBP3ΔTM and *Pa*PBP3ΔTM, respectively (**E**,**F**). The reference experiments with addition of DMSO only are displayed in gray, whereas the graphs for complexes with aztreonam and AIC499 are colored green and gold, respectively.

Direct comparison between AIC499 and aztreonam acyl-enzyme intermediates (Figure 7) reveals significant differences at the extremities of the molecules, whereas the core structure including the amino-thiazole moiety and the carboxyl group in the linker are conserved. Specifically, note the dramatic increase in contact area because of the R^1^ head group of AIC499 (panels A and C), which mostly participates in hydrophobic interactions. Conversely, replacement of the terminal sulfonate by sulfate (panels B and D) is likely to provide additional freedom for electrostatic interactions, but an equally relevant modification in this region may be the additional methyl substituent (R^3^); in all AIC499 complexes investigated, one of these methyl groups contributes to the hydrophobic cluster orchestrated by the phenyl ring, effectively linking both branches of the molecule. Irrespective of the differences outlined above, binding of both compounds entails a significant increase in thermal stability of PBP3. In the case of *E. coli* PBP3 (Figure 7E), the midpoint of thermal unfolding (*T*_m_) determined via differential scanning fluorometry (DSF) increased from 58.6 to 63.4 °C for aztreonam and to 65.4 °C for AIC499, whereas for the *P. aeruginosa* protein (Figure 7F) the value rises from 46 to 54 °C for both complexes. The temperature-dependent shifts in the 350 nm/330 nm fluorescence ratio were closely mirrored by increases in turbidity (Figure A1), supporting the view that they do not merely reflect local changes in the environment of aromatic residues, but they correspond to real unfolding transitions.

**Figure A2:**
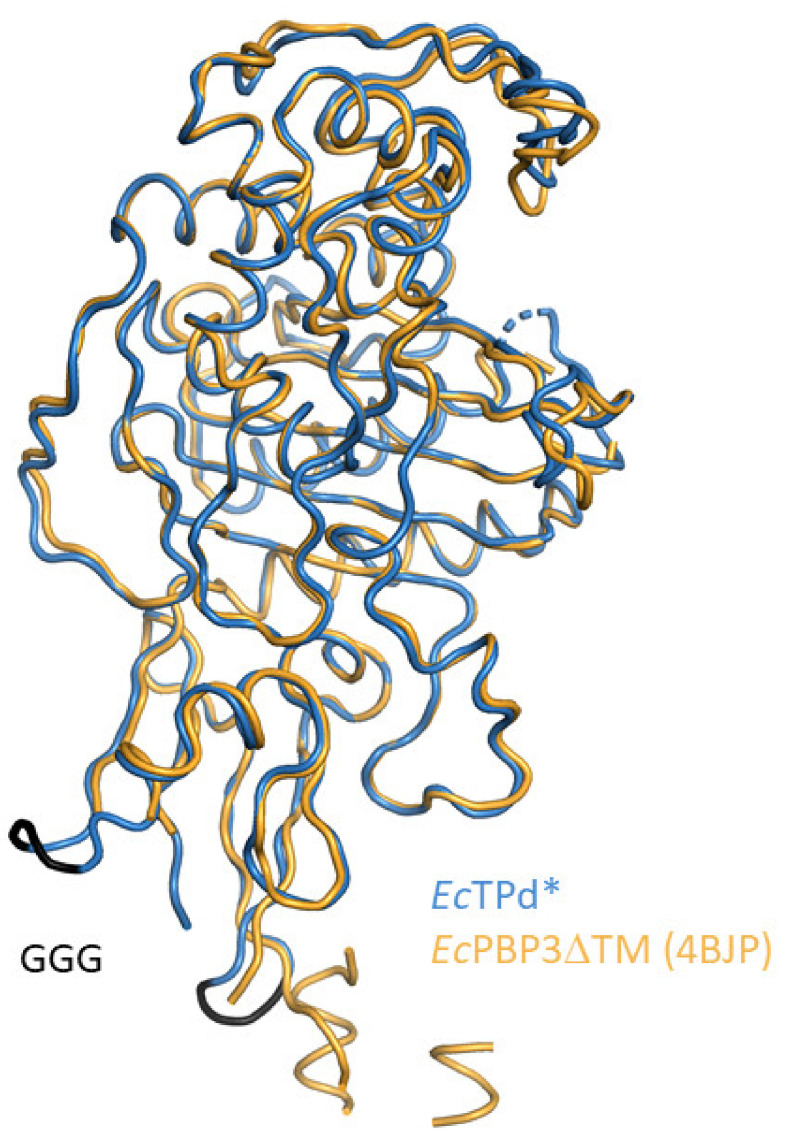
Superposition of the X-ray structures of the *Ec*TPd* protein introduced in this study (dark blue) and *Ec*PBP3ΔTM published previously (PDB entry 4BJP, gold), demonstrating the similar coverage of ordered structure. The GGG linkers replacing the deleted segments in *Ec*TPd* are colored black.

Our crystallographic investigation revealed a considerably higher flexibility of *Ec*PBP3 when compared with its *P. aeruginosa* orthologue, particularly in the extended *N*-terminal portions of the head and anchor subdomains. This observation is consistent with data published previously on the *E. coli* apo form [13]. The reasons for this difference are unclear but are likely to reflect differences in the properties of one or more interaction partners in the multiprotein complex (the divisome) that PBP3 is chiefly involved in. To circumvent the drawbacks associated with enhanced dynamics in crystallographic studies, we introduced a new truncated construct for the *E. coli* protein, yielding crystals with significantly improved diffraction quality. Notably, our design differs from the one published previously by Bellini et al. (PDB entry 6HZQ [38]). While those authors used a straightforward *N*-terminal truncation, keeping residues 234-588, we decided to include the linker subdomain in the construct because it intimately interacts with the core TPase fold and is unlikely to be affected by enhanced dynamics. Since the linker subdomain is discontinuous, this involved removal of two internal sequence segments, in addition to the far *N*-terminus. Indeed, the structural data thus obtained for our truncated *Ec*PBP3 variant (*Ec*TPd*) allowed us to trace the entire linker region, which is the vast majority of *N*-terminal residues included in the previous structure of soluble *Ec*PBP3ΔTM (PDB entry 4BJP; refer to Figure A2 for a superposition), yet avoiding the negative impact of excessive dynamics on crystal packing and diffraction quality.

Notably, the presence or absence of the linker subdomain has a significant influence on adjacent structural features of the TPase fold. For example, we were able to resolve the region between P279 and R297, which is not included in the previous *Ec*TPd structure, presumably because it is stabilized by interactions with the linker region. In addition, residues R297-T300 adopt a non-native extended conformation in PBP entry 6HZQ, instead of the partly helical structure observed in our model. This local restructuring appears to propagate into other parts of the protein, especially the segment connecting helices α6 and α8; the region G392-W407 is disordered in PDB entry 6HZQ, whereas in our structure it was modeled completely, including most of the side chains. Notably, our structure of *Ec*TPd* is consistent with previous data for soluble *Ec*PBP3 (PBP entry 4BJP), confirming it closely reflects the native fold. In the vicinity of the active site the most notable differences are found in the β5-α11 region; it is entirely ordered and orientated towards the active site in PBP entry 6HZQ, resembling what is commonly found after ligand binding. The reasons for this discrepancy are unclear, given that this region is not restrained by lattice contacts. In contrast, the notoriously flexible parts of the β2b-β2c-β2d region and the β3-β4 hairpin appear more similar between the two *Ec*PBP3 structures. Taken together, these observations support the view that the linker region contributes significantly to the stable fold of the TPd of *Ec*PBP3 and, by extension, its orthologues in other species. Therefore, it appears advisable not to exclude this segment when designing truncated constructs for biophysical applications.

Furthermore, the utility of this new construct is not limited to crystallography. In this context, we showed that the *Ec*TPd* protein (44 kDa) is suitable for NMR experiments, yielding very good signal dispersion in the 2D ^1^H-^15^N TROSY-HSQC spectrum on a spectrometer with modest field strength (700 MHz). In contrast, acquisition of good quality spectra of *Ec*PBP3ΔTM (60 kDa) is challenging because the dynamic n-PBd gives rise to a 2D ^1^H-^15^N TROSY-HSQC spectrum with severe resonance overlap (Figure 2C) and an unfavorably longer correlation time. Sequence specific assignment of the 2D ^1^H-^15^N TROSY-HSQC spectrum for the *Ec*TPd* protein is ongoing and should facilitate fragment-based screening programs.

Regarding the *Pa*PBP3 orthologue, the newly identified crystal forms described in this paper expand the repertoire of structural information on the apo protein, allowing for a more reliable assessment of protein dynamics and the impact of lattice interactions. For example, the β3-β4 region, while clearly showing enhanced dynamics, is traced to a larger extent than described previously, indicating a β-hairpin conformation that protrudes from the TPase fold. Previous structures of apo-*Pa*PBP3ΔTM, e.g., PDB entry 6HZR, were lacking coordinates for the β3-β4 loop, and it was assumed that this region is only stabilized upon acylation of the catalytic serine. For the β5-α11 region electron density suggests a high degree of conformational freedom with our crystal form 1 and PDB entry 6HZR representing two extremes of the conformational space sampled, i.e., the β5-α11 loop bends in opposite directions.

The structural data presented here may guide future efforts to further improve the properties of AIC499. One exciting option, inspired by the remarkable U-shape of protein-bound AIC499, is introduction of a covalent linkage or salt bridge between R^1^ and R^3^, resulting in a cyclized compound with enhanced rigidity; such a modification may strongly affect PBP3 affinity and β-lactamase stability as well as impact pharmacokinetics. Given the unique role of PBPs in bacterial cell wall homeostasis, further development of compounds targeting these proteins is certainly warranted.

## Figures and Tables

**Table 1:** Data collection and refinement statistics of PBP3 structures reported in this paper (cf, crystal form). Values in parentheses refer to the highest-resolution shell.

	*Ec*TPd*	*Ec*TPd*: AIC499	*Ec*PBP3 ΔTM: AIC499	*Pa*PBP3 ΔTM (cf 1)	*Pa*PBP3 ΔTM (cf 2)	*Pa*PBP3 ΔTM (cf 3)	*Pa*PBP3 ΔTM: AIC499
PDB entry	7ONO	7ONN	7ONW	7ONX	7ONY	7ONZ	7ONK
**Data collection**							
Beamline	DESY P11	DESY P11	ESRF ID23	DESY P11	EMBL P13	EMBL P13	DESY P11
Wavelength [Å]	1.0332	1.0332	0.97242	1.0332	0.9999	0.9999	1.0332
Space group	P 6_2_ 2 2	P 6_2_ 2 2	P 6_4_ 2 2	C 1 2 1	C 1 2 1	C 1 2 1	P 2_1_ 2_1_ 2_1_
Cell dimensions							
a, b, c [Å]	109.3, 109.3, 143.2	110.4, 110.4, 142.1	106.9, 106.9, 285.8	110.6, 82.2, 91.4	104.1, 125.0, 74.2	151.5, 37.5, 82.8	81.0, 91.1, 148.4
α, β, γ [°]	90, 90, 120	90, 90, 120	90, 90, 120	90, 116.3, 90	90, 122.5, 90	90, 112.6, 90	90, 90, 90
Resolution range [Å]	47.74–2.30 (2.60–2.30)	47.80–1.92 (2.22–1.92)	48.64–2.70 (3.03–2.70)	44.40–2.16 (2.34–2.16)	39.55–1.77 (1.97–1.77)	40.75–1.86 (2.09–1.86)	46.92–1.73 (1.90–1.73)
CC_1/2_ [%]	99.9 (84.0)	99.9 (78.3)	99.9 (79.3)	99.6 (47.1)	99.8 (64.1)	99.8 (77.2)	99.9 (62.4)
*R*_meas_ [%]	10.3 (145.6)	6.6 (160.2)	10.1 (121.1)	11.1 (101.5)	7.0 (115.9)	7.1 (96.6)	9.4 (92.5)
I/σ	20.7 (2.4)	24.2 (2.3)	15.4 (2.0)	7.9 (1.4)	13.9 (1.8)	12.3 (1.6)	10.9 (1.6)
Completeness [%] ^a^	40.8 (6.7)	45.7 (6.5)	51.1 (9.0)	66.6 (15.4)	55.5 (10.4)	51.8 (8.8)	74.2 (15.3)
Ellips. Compl. [%] ^b^	93.9 (74.6)	95.0 (74.3)	92.2 (76.1)	91.4 (57.8)	70.4 (4.4)	74.8 (3.6)	95.9 (64.8)
**Refinement**							
Resolution range [Å]	47.74–2.30	47.80–1.92	48.64–2.70	44.40–2.16	39.55–1.77	40.75–1.86	46.92–1.73
No. unique reflections	9457	18221	13994	26398	42915	19031	85337
No. protein atoms	3031	3022	3516	3609	3841	3668	7803
No. ligand atoms	5	135	71	32	118	12	260
No. water molecules	16	60	6	146	274	134	750
*R*_work_ [%]	23.00	22.33	23.35	18.17	17.98	20.91	17.34
*R*_free_ [%]	28.20	26.93	27.07	21.52	21.34	25.85	21.52
RMSD							
Bond lengths [Å]	0.001	0.005	0.002	0.003	0.004	0.003	0.007
Bond angles [°]	0.403	1.031	0.672	0.579	0.666	0.601	1.037
Mean B factor [Å^2^]	50.49	41.23	64.13	42.63	34.16	29.60	23.60
Ramachandran plot							
Favored [%]	95.52	93.98	95.01	95.98	96.79	96.30	97.72
Allowed [%]	4.48	6.02	4.99	4.02	3.21	3.70	2.28
Outliers [%]	0	0	0	0	0	0	0

^a^ Conventional definition using spherical shells. ^b^ Calculated with respect to an ellipsoidal portion of reciprocal space fitted to the cut-off surface, as defined in STARANISO. Low values for *Pa*PBP3ΔTM cf2 and cf3 are a consequence of a rugged cut-off surface complicating the determination of a meaningful ellipsoid.

## Appendix A

**Table A1:** Overview of the distances between atoms of AIC499 (center) and *Pa*PBP3ΔTM (chain B, left) or *Ec*TPd* (right). The type of interaction as well as the functional group which the respective AIC499 atom belongs to are given in parentheses. See Figure 1 for the nomenclature of AIC499 atoms. Hydrogen bonds are included with donor-acceptor distances ranging from 2.3 to 3.2 Å, and hydrophobic and aromatic interactions are listed for distances between 3.0 and 4.5 Å. For distances differing between both conformers, the smaller value is given.

*Pa*PBP3ΔTM		AIC499	*Ec*TPd*	
*Atom*	*Distance [Å]*	*Atom*	*Distance [Å]*	*Atom*
/	/	C2 (R^1^)	3.6 (hydrophobic)	Y541 C_ε2_
V333 C_γ1_	3.5 (hydrophobic)	C4 (R^1^)	/	/
V333 C_γ2_	3.6 (hydrophobic)	C11 (R^1^)	3.7 (hydrophobic)	V344 C_γ1_
Y532 C_ε2_	3.8 (aromatic)	C13 (R^1^)	4.2 (aromatic) ^b^	Y540 C_ε2_
T487 C_β_	3.5 (hydrophobic)	C14 (R^1^)	4.2 (hydrophobic)	T497 C_β_
F533 C_β_	3.7 (aromatic) ^a^	C15 (R^1^)	4.0 (aromatic)	Y541 C_δ1_
T487 C_β_	4.3 (hydrophobic)	C18 (R^5^)	/	/
/	/	C19 (R^5^)	4.1 (hydrophobic)	Y511 C_ε2_
R489 N_η2_	3.0 (H-bond)	O20 (R^5^)	/	/
T487 O	3.2 (H-bond)	N23	/	/
R489 N	2.8 (H-bond)	N26 (R^4^)	3.0 (H-bond)	K499 N
Y409 C_δ1_	3.8 (aromatic)	C27 (R^4^)	3.6 (aromatic)	Y419 C_ε1_
A488 C_α_	3.8 (hydrophobic)	C27 (R^4^)	3.6 (hydrophobic)	A498 C_α_
E291 O_ε1_	2.5 (H-bond)	N28 (R^4^)	2.3 (H-bond)	E304 O_ε1_
R489 O	2.5 (H-bond)	N28 (R^4^)	2.8 (H-bond)	K499 O
/	/	N28 (R^4^)	3.1 (H-bond)	K499 N
/	/	C30 (R^4^)	4.4 (aromatic)	F417 C_δ1_
G293 C_α_	3.8 (hydrophobic)	C30 (R^4^)	3.8 (hydrophobic)	G306 C_α_
N351 N_δ2_	2.8 (H-bond)	O32	3.0 (H-bond)	N361 N_γ2_
T487 O	2.8 (H-bond)	N33	3.2 (H-bond)	T497 O
V333 C_γ2_	3.6 (hydrophobic)	C36 (R^3^)	/	/
T487 C_β_	3.8 (hydrophobic)	C36 (R^3^)	/	/
V333 C_γ2_	3.8 (hydrophobic)	C37 (R^3^)	3.6 (hydrophobic)	V344 C_γ1_
S349 C_β_	4.3 (hydrophobic)	C37 (R^3^)	/	/
S294 O_γ_	2.7 (H-bond)	N38 (β-lactam ring)	/	/
S349 O_γ_	2.8 (H-bond)	N38 (β-lactam ring)	/	/
S294 N	2.7 (H-bond)	O40	2.5 (H-bond)	S307 N
T487 N	2.9 (H-bond)	O40	3.2 (H-bond)	T497 N
S294 O_γ_	3.0 (H-bond)	O41 (R^2^)	3.0 (H-bond)	S307 O_γ_
K484 N_ζ_	2.9 (H-bond)	O44 (R^2^)	2.7 (H-bond)	T497 O_γ_
S485 O_γ_	2.4 (H-bond)	O44 (R^2^)	/	/
T487 O_γ_	2.5 (H-bond)	O45 (R^2^)	3.1 (H-bond)	K494 N_ζ_
T487 N	2.9 (H-bond)	O45 (R^2^)	3.1 (H-bond)	T495 O

^a^ Inferred from the C_β_ position, although the phenyl group is not resolved in the electron density. ^b^ An even shorter distance of 4.0 Å is found between AIC499 C12 (R^1^) and *Ec*TPd* Y540 C_ζ_.

**Table A2:** Overview of the distances between atoms of aztreonam and *Pa*PBP3 (PDB entry 3PBS [11]), with type of interaction given in parentheses. Aztreonam atoms are named in accordance to PDB entry 3PBS, and equivalent atoms of AIC499 together with the respective functional groups are shown in parentheses. Hydrogen bonds are included with donor-acceptor distances ranging from 2.3 to 3.2 Å, and hydrophobic and aromatic interactions are listed for distances between 3.0 and 4.5 Å.

*Pa*PBP3	*Distance [Å]*	Aztreonam
*Atom*		*Atom*
T487 C_γ2_	3.6 (hydrophobic)	C28 (C4; R^1^) ^c^
Y532 C_ε2_	4.4 (hydrophobic)	C28 (C13; R^1^) ^c^
Y503 C_ε1_	3.7 (hydrophobic)	C28 (C13; R^1^) ^c^
F533 C_β_	3.9 (hydrophobic)	C27 (C15; R^1^) ^c^
R489 N_ε_	3.1 (H-bond)	O31 (O20; R^5^)
A488 C_α_	4.2 (hydrophobic)	C25 (C25; R^4^)
Y409 C_δ1_	3.9 (aromatic)	C26 (C27; R^4^)
A488 C_α_	3.9 (hydrophobic)	C26 (C27; R^4^)
E291 O_ε1_	2.9 (H-bond)	N16 (N28; R^4^)
G293 C_α_	4.0 (hydrophobic)	C25 (C30; R^4^)
N351 N_δ2_	2.9 (H-bond)	O10 (O32)
T487 O	3.0 (H-bond)	N13 (N33)
T487 C_β_	4.3 (hydrophobic)	C18 (C35; R^3^)
S294 C_β_	4.4 (hydrophobic)	C18 (C35; R^3^)
V333 C_γ1_	3.6 (hydrophobic)	C7 (C36; R^3^)
S294 O_γ_	3.1 (H-bond)	N12 (N38; β-lactam ring)
S349 O_γ_	3.0 (H-bond)	N12 (N38; β-lactam ring)
G293 C_α_	4.5 (hydrophobic)	C20 (C39)
S294 N	3.0 (H-bond)	O9 (O40)
T487 N	2.8 (H-bond)	O9 (O40)
K484 N_ζ_	3.1 (H-bond)	O34 (O44; R^2^)
S485 O_γ_	2.7 (H-bond)	O34 (O44; R^2^)
T487 O_γ_	2.6 (H-bond)	O33 (O45; R^2^)
T487 N	3.2 (H-bond)	O33 (O45; R^2^)

^c^ As R^1^ moiety aztreonam only contains two methyl groups.
