# Correction: Freischem et al. Interaction Mode of the Novel Monobactam AIC499 Targeting Penicillin Binding Protein 3 of Gram-Negative Bacteria. *Biomolecules* 2021, *11*, 1057

**DOI:** 10.3390/biom12010005

**Published:** 2021-12-21

**Authors:** Stefan Freischem, Immanuel Grimm, Arancha López-Pérez, Dieter Willbold, Burkhard Klenke, Cuong Vuong, Andrew J. Dingley, Oliver H. Weiergräber

**Affiliations:** 1 Institute of Biological Information Processing (IBI-7: Structural Biochemistry) and Jülich Centre for Structural Biology (JuStruct), Forschungszentrum Jülich, 52425 Julich, Germany; s.freischem@fz-juelich.de (S.F.); d.willbold@fz-juelich.de (D.W.); a.dingley@fz-juelich.de (A.J.D.); 2 Institut für Physikalische Biologie, Heinrich-Heine-Universität Düsseldorf, 40225 Dusseldorf, Germany; 3 AiCuris Anti-Infective Cures AG, 42117 Wuppertal, Germany; immanuel.grimm@Aicuris.com (I.G.); arancha.lopez@aicuris.com (A.L.-P.); burkhard.klenke@gmail.com (B.K.); cuong.vuong@aicuris.com (C.V.); 4 Centre for Bacterial Cell Biology, Biosciences Institute, Newcastle University, Newcastle upon Type NE2 4AX, UK

In the original article [1], there was a mistake in Figure 1 as it was published. The double bond connecting N-23 and C-24 was drawn with the wrong configuration. The corrected Figure 1 appears below. The authors apologize for any inconvenience caused and state that the scientific conclusions are unaffected. The original article has been updated.

## Figures and Tables

**Figure 1:**
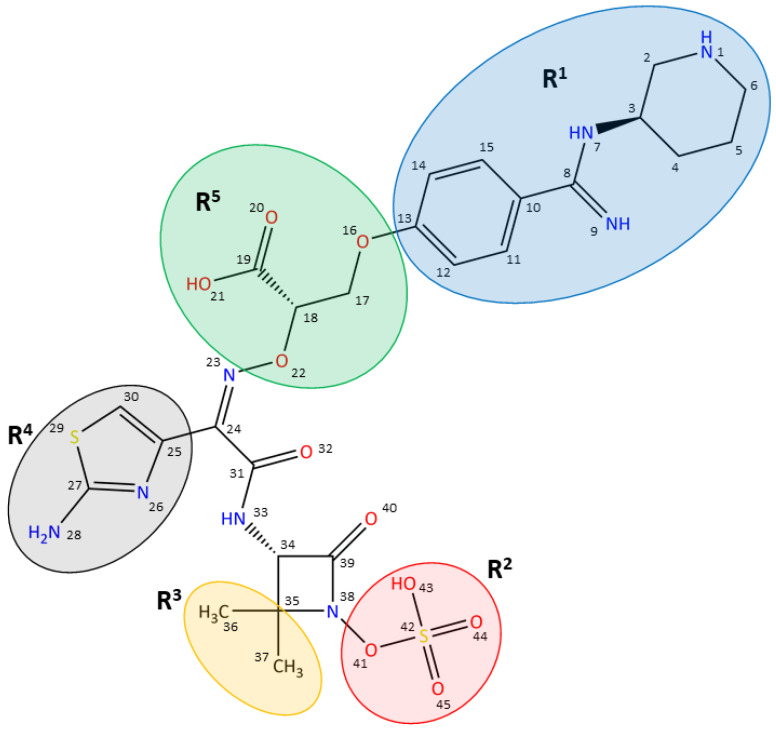
Structure of the monobactam AIC499, obtained by iterative optimization. The relevant functional groups are marked in color: benzamidine-based head group (R^1^), blue; β-lactam N-1 position (R^2^), red; β-lactam C-4 position (R^3^), orange; amino-thiazole (R^4^), gray; linker (R^5^), green.
